# Detecting falsified oral contraceptives by visual assessment and diffuse reflectance spectroscopy (350–2500 nm): the need for supplementing traditional pharmacopeia techniques and the public health implications

**DOI:** 10.1016/j.heliyon.2022.e10837

**Published:** 2022-10-01

**Authors:** David Jenkins, Cherif Diallo, Michael Payne

**Affiliations:** Product Quality and Compliance, FHI 360, 2810 Meridian Parkway, Suite 160, Durham, NC 27713, USA

**Keywords:** Falsified pharmaceutical, Rapid screening, Oral contraceptives, Public health

## Abstract

**Objectives:**

Substandard and falsified pharmaceuticals can present a major health risk, particularly for low- and middle-income countries. In a Sub-Saharan African market, United States Agency for International Development (USAID) staff found an oral contraceptive product (0.15 mg levonorgestrel/0.03 mg ethinyl estradiol) labeled with a logo (and brand name) visually matching one historically used by USAID but purportedly manufactured by an unauthorized source. Additional assessment was conducted to determine if more evidence of falsification was present to better understand the public health impact.

**Study design:**

Relative to a control, the suspect sample was assessed visually for tablet features and with United States and International Pharmacopeia methods for levonorgestrel/ethinyl estradiol tablets. Diffuse reflectance spectra (350–2500 nm) were collected to further characterize the sample.

**Results:**

Although the suspect sample aligned with pharmacopeia tests, visual examination of tablet characteristics and diffuse reflectance spectroscopy (350–2500 nm) assessments supported the tablets were not the same as control samples, confirming the suspect sample was falsified. These results raised concerns for the overall regulatory oversight available for the market with uncertainty regarding the true clinical bioequivalence levels (although adequate dissolution levels were observed).

**Conclusions:**

Comprehensive characterization of suspect pharmaceuticals from the field can often be required depending on the nature of the sample and can have dramatic implications for understanding the public health risks to the end user within the local market. Simple visual assessment and spectroscopic techniques to screen a sample can help to supplement traditional pharmacopeia approaches.

**Implications:**

Proper characterization of suspect pharmaceuticals is necessary to best understand their potential public health impact. Situations can occur where traditional pharmacopeial techniques may not adequately characterize a sample. Visual assessments and diffuse reflectance spectroscopy can be supplemented to provide a more holistic analysis.

## Graphical abstract

**Figure:**
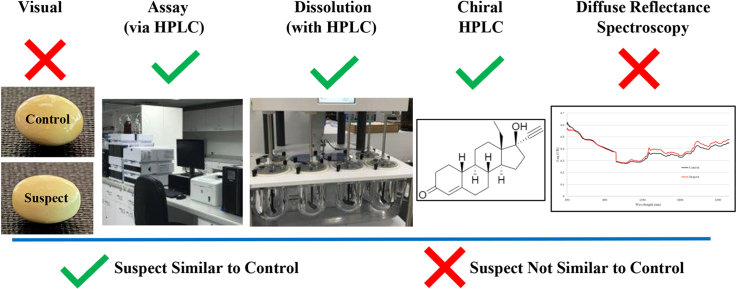

## 1 Introduction

According to the World Health Organization, approximately 10% of medicines in low- and middle-income countries (LMICs) may be substandard or falsified [1]. The main reasons for poor quality medicines are inadequate manufacturing from the legitimate source, degradation, and/or product falsification [2]. Prominent examples of poor-quality medicines (substandard/falsified) have been reported for various antimalarials [3, 4]. Quality problems encompassing substandard and/or falsification have also occurred for antibiotics [5], emergency contraceptives [6], and medications for diabetes [7]. Poor quality medicines raise health risks for end-users [8], while increasing the financial burden on health care systems [9].

Commonly, poor quality medicines are assessed with more traditional pharmacopeia methods (often HPLC based). A variety of alternative techniques are available that involve spectrometers and devices that are often more portable than traditional techniques [10, 11]. The GPHF-Minilab incorporates thin layer chromatography (TLC) and different physical techniques that can be transported to more remote locations and has been used in various assessments [12, 13]. Instruments using near infrared (NIR) and Raman spectroscopy have been used to assess medicine quality [14, 15], also coupled with other techniques (i.e., visual, colorimetry) [16].

This work presents the characterization of oral contraceptives, specifically tablets containing both levonorgestrel and ethinyl estradiol, found in the market that were suspected of being falsified (purposely mislead the identity, composition, or source of product [1, 17]). To promote trust and continuity with oral contraceptives supplied by the United States Agency for International Development (USAID) over the last several decades, a specific logo is provided on the outer packaging of the product manufactured by specific contract sources (with the product’s brand name) for various country programs. While in a Sub-Saharan Africa country, USAID staff observed a box of oral contraceptives that showed the USAID logo and product brand name but indicated a different manufacturing source. Levonorgestrel and ethinyl estradiol tablets provide contraceptive effectiveness through various mechanisms, such as preventing ovulation and impeding sperm entry through cervical mucus thickening [18], and have narrow ranges of therapeutic effectiveness making bioavailability/bioequivalence important aspects for consideration [19, 20]. Due to concerns for public safety, potential bioequivalence concerns, product registration, and misuse of USAID’s logo, the sample was acquired to determine if further evidence of falsification could be found and to better understand the potential health impact. With chromatographic pharmacopeia tests used for levonorgestrel and ethinyl estradiol tablets as a core portion of the characterization, this analysis assesses the impact of including visual and spectroscopic techniques to supplement traditional pharmacopeia approaches when investigating suspect pharmaceuticals which can have important public health consequences.

## 2 Materials and methods

### 2.1 Samples

The oral contraceptives are comprised of 21 active tablets and 7 hormone free tablets per blister pack with levonorgestrel (0.15 mg) and ethinyl estradiol (0.03 mg) in each active tablet. One blister pack (with outer box) of suspect sample was able to be purchased from a local pharmacy in Sub-Saharan Africa and made available for testing. Further details disclosing product brand name, manufacturer, and purchasing location of the suspect sample will not be provided for legal reasons but have been provided to USAID for investigation with the authentic manufacturer and Sub-Saharan African country’s regulatory authority. Multiple blister packs of authentic product (from the same lot) were obtained from USAID’s retain supply as a control sample.

### 2.2 Visual characterization

Digital images of the front and back of both outer boxes and the blister packs were captured for the control and suspect samples and provided to USAID for review (not provided here to protect the manufacturer). From these images, the general features of the packaging and tablets for the suspect sample was visually compared to the control samples.

### 2.3 Chemical testing

Chemical testing for quantifying active ingredient and dissolution was based on the USP monograph for Levonorgestrel and Ethinyl Estradiol tablets [21]. With limited sample, five tablets were tested for active ingredient quantification and three tablets for dissolution (less than specified in the monograph). Testing this amount for content and dissolution allowed sample characterization for these parameters while conserving sample for other types of testing. HPLC grade acetonitrile was obtained from Honeywell Burdick & Jackson (Muskegon, MI, USA). HPLC grade methanol was obtained from both Honeywell Burdick & Jackson (Muskegon, MI, USA) and LabChem (Zelienople, PA, USA). Purified water (18 MΩ·cm resistivity) was provided with a Hydro Picopure® system (Durham, NC, USA). Ethanol (200 proof, USP grade) was obtained from Koptec (King of Prussia, PA, USA). Tween 80 N.F. grade (polysorbate 80) was purchased from J.T. Baker Avantor Performance Materials (Center Valley, PA, USA). γ-Cyclodextrin (>99%) was obtained from Tokyo Chemical Industry (Tokyo, Japan). Levonorgestrel, norgestrel, and ethinyl estradiol reference standard (RS) were purchased from the United States Pharmacopeia (USP, Rockwell, Maryland, USA). High performance liquid chromatography (HPLC) was conducted with an Agilent HP1200 operated by ChemStation software with a G1322A degasser, G1329A autosampler, G1311A quaternary pump, G1316A column thermostat, G1315B diode array UV detector, and G1321A fluorescence detector (Agilent Technologies, Santa Clara, CA, USA). Active ingredient quantification and dissolution was conducted with Phenomenex guard columns comprised of a KJ0-4282 cartridge holder with C8 cartridges 4-mm x 3-m with 5 μm particle size P/N-AJ04290 (Torrance, CA, USA). Tablet weights were collected (Mettler AT20 balance) from the samples used for active ingredient quantification.

Active ingredient quantification was conducted with HPLC using a Phenomenex Luna C8 4.6-mm x 15-cm with L7 packing of 5 μm 100 Å particle size (P/N-00F-4249-E0, S/N-651521-9, Torrance, CA, USA). The chromatography used an isocratic mobile phase of acetonitrile: methanol: water 35:15:45 v/v under ambient temperature with a 1 mL/min flow rate, 215 nm detection, and 50 μL injection. Standards contained both levonorgestrel RS (0.015 mg/mL) and ethinyl estradiol RS (0.003 mg/mL) in mobile phase. Samples were prepared by placing 1 tablet in a 40 mL centrifuge tube with 10 mL of mobile phase, sonicating (30 min), mechanically shaking (20 min), and centrifuging (2000–3000 RPM for 10–15 min) to obtain the supernatant. Individual tablets were quantified as % label claim (LC) per active.

Dissolution was conducted with a VK 7000 Varian apparatus (VanKel Technology Group, Cary, NC, USA), where each vessel contained 500 mL of medium, polysorbate 80 (5 μg/g) in water, at 37 °C with paddles operating at 75 RPM. An aliquot of ∼15 mL was removed 60 min after tablets were dropped in separate vessels. Final working standards contained both levonorgestrel RS (0.0003 mg/mL) and ethinyl estradiol RS (0.00006 mg/mL) in dissolution medium (containing ∼0.3% volume of ethanol). Standards and samples were analyzed with HPLC using a Phenomenex Luna C8 4-mm x 15-cm with L7 packing of 5 μm 100 Å particle size (P/N-00F-4040-D0, S/N-263359-1, Torrance, CA, USA). The chromatographic conditions used an isocratic mobile phase of acetonitrile:water (60:40) v/v operating under ambient temperature with a 1 mL/min flow rate, 247 nm UV detection for levonorgestrel, 285 nm excitation/310 nm emission fluorescence detection for ethinyl estradiol, and 100 μL injection. Dissolution (%Q) was calculated for individual tablets based on the percent active ingredient released relative to the theoretical amount in the tablet.

### 2.4 Chiral chromatography

To provide evidence the tablets contained levonorgestrel (not norgestrel), chiral chromatography was conducted [6, 22]. Norgestrel and levonorgestrel RS solutions were prepared each at ∼12 μg/mL in diluent (methanol:water 80:20). Control and suspect samples were prepared each at ∼60 μg/mL (levonorgestrel), where one crushed tablet was mixed with 2.5 mL of diluent and heated at 60 °C for 10 min (with periodic shaking). Standards and sample were filtered through 0.45 μm PTFE (Acrodisc R25 P/N 4219T, Pall Corporation, Port Washington, NY, USA) before injection. The chromatography used an isocratic mobile phase (1% γ-cyclodextrin (aq.):methanol 50:50) and ODS Hypersil column 150 × 4.6 mm with 5 μm particle size (P/N 30105, S/N 12171883Q3, Thermo Fisher Scientific, Waltham, MA, USA), at ambient temperature with a 1.5 mL/min flow rate, 242 nm UV detection, and a 50 μL injection.

### 2.5 Diffuse reflectance spectroscopy

Like other approaches [23], diffuse reflectance spectra (dependent on chemical/physical properties) of tablets were obtained using a LabSpec® 5000 spectrometer (350–2500 nm, tungsten halogen lamp, InGaAs detector, 10 nm resolution), with Muglight, sample tray adapter, and spectralon puck accessories (Malvern Panalytical Ltd., UK). The spectrometer uses IndicoPro software (Malvern Panalytical Ltd., UK) for data collection, with GRAMS/AI (and IQ Predict) software (ThermoFisher, Waltham, MA, USA) for analysis. Spectra were collected using the Muglight accessory with attached sample tray adapter. Background scans were collected with a 1-inch diameter spectralon puck placed within the sample tray adapter. For samples, each tablet was placed in the center of the sample tray adapter and covered with a 3.5-inch spectralon puck, where three 50 scan subsets were collected with ∼45° rotation per subset. Sample spectra were processed by GRAMS/AI (with IQ Predict) through a discriminant identification method (providing a Mahalanobis distance (D) via a principal component analysis (PCA) algorithm) prepared for routine monitoring of the OC brand name product provided by USAID.

To build the method, scans from 78 different active tablets (across 15 lots previously tested for active ingredient quantity and dissolution [21]) of USAID’s supplied product were loaded as references into GRAMS/AI (with the same processing parameters used previously [23]). Through the PCA algorithm, 10 factors were used to construct the method loaded into GRAMS IQ Predict to provide D’s of sample spectra. With 78 reference spectra and 10 factors, a D_max_ threshold of 4.73 was calculated at a 95% confidence level [23, 24]. To evaluate the level of discernability of the method, spectra of other active tablets (n = 27) across the same 15 lots used to construct the method were assessed as positive controls (PC) and provided D’s from 0.5 to 2.2. Levonorgestrel and ethinyl estradiol tablets from two other manufacturers (brand names not provided) were used as negative controls (NC). Although active NC tablets provided compliant results for active ingredient quantity and dissolution [21], spectra from active tablets generated D values greater than the D_max_ of 4.73 (NC1, AVG D = 70.1, n = 10; NC2, AVG D = 384.4, n = 10). The results from the positive/negative controls showed the method could discriminant tablets manufactured by other sources relative to the reference spectra. With this method, five active tablets from control and suspect samples were assessed for Mahalanobis distances relative to reference spectra.

## 3 Results

### 3.1 Visual assessment

**Figure 1:**
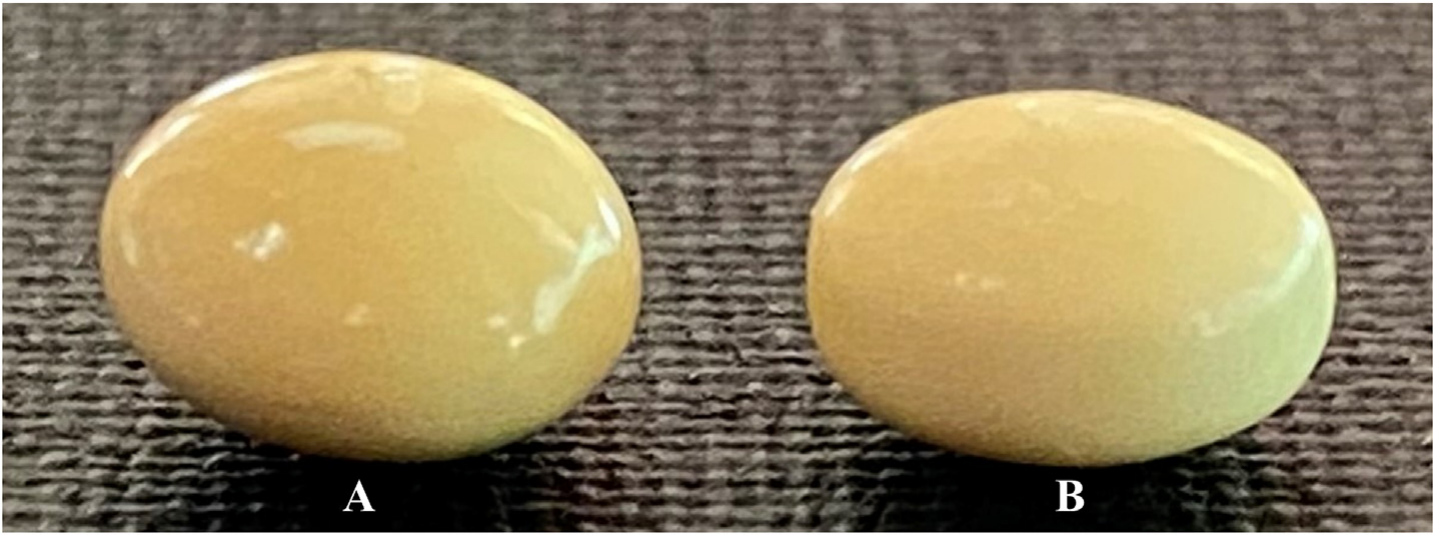
Example tablets of an authentic control (A) and a suspect sample (B).

Visual differences first identified the suspect sample. A replica of USAID’s logo historically used for their brand was visible on the box but stated a different manufacturer than used by USAID. Further inspection of the box and blister packs (i.e., color and text) found other distinctions from the authentic product. An image of example tablets are provided in Figure 1a for the control sample and 1b for the suspect sample. Although difficult to assess from an image, the suspect samples appeared smaller, with a more cylindrical shape (more vertically oriented sides) relative to the more rounded nature (i.e., oval shape) of the control. Furthermore, suspect samples had a lighter yellow color relative to authentic samples.

### 3.2 Tablet weights, API quantification and dissolution

Table 1 summaries the results for tablet weights, active ingredient quantity, dissolution, and diffuse reflectance spectroscopy. The suspect sample had a ∼9.5% lower tablet weight relative to the authentic (consistent with smaller tablets). Levonorgestrel and ethinyl estradiol content was slightly lower relative (but comparable) to the control, but the variance was greater in the suspect sample. Dissolution levels were comparable between the sample and the authentic. Although strict compliance cannot be claimed because of limited tablets tested, the active ingredient content and dissolution levels are consistent with USP criteria.

**Table 1:** Test results from the control and suspect sample.

Testing Parameter	Authentic Control Sample	Suspect Sample
Tablet weights (mg) – average of 5 tablets	90.84 (2.8% RSD)	82.19 (3.6% RSD)
%LC Content of EE - average of 5 tablets (USP requirement of 90.0–110.0% of LC)	98.1 (1.2% RSD)	95.5 (3.8% RSD)
%LC Content of L - average of 5 tablets (USP requirement of 90.0–110.0% of LC)	99.0 (0.9% RSD)	97.8 (4.8% RSD)
%Q EE Dissolution - average of 3 tablets (USP minimum requirement = 60%)	88.0 (2.3% RSD)	91.3 (2.5% RSD)
%Q L Dissolution - average of 3 tablets (USP minimum requirement = 60%)	91.7 (2.3% RSD)	90.7 (1.3% RSD)
Mahalanobis Distance (D) of Diffuse Reflectance Spectra - average of 5 tablets (D_max_ = 4.73)	2.2 (23.4% RSD)	44.3 (32.4% RSD)

RSD = relative standard deviation; LC = label claim; EE = ethinyl estradiol; L = levonorgestrel.

### 3.3 Assessment of levonorgestrel and dextronorgestrel enantiomers

**Figure 2:**
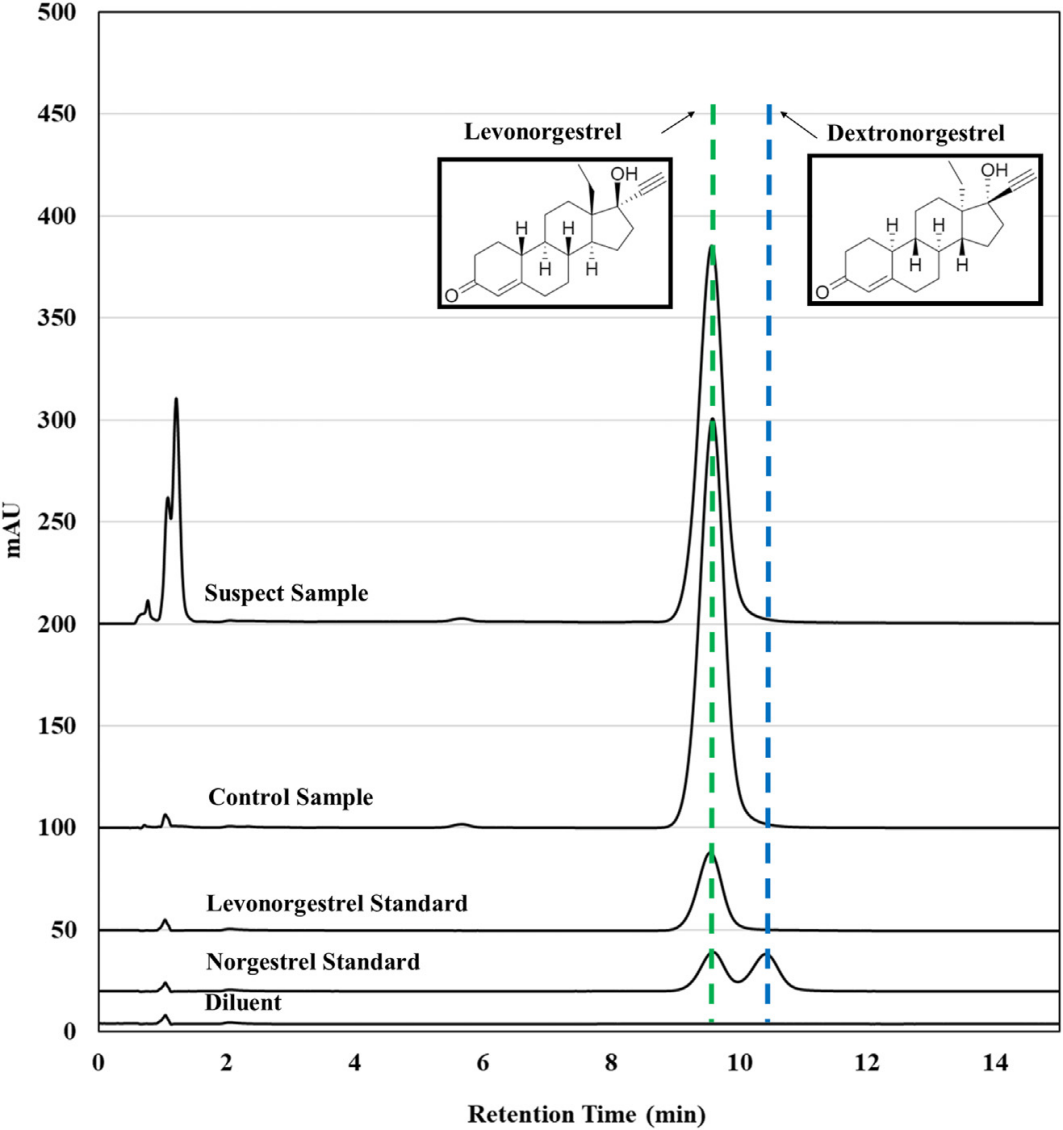
Chiral chromatographic results to distinguish levonorgestrel and dextronorgestrel from within the control and suspect samples.

From the HPLC analysis for content and dissolution, identification of ethinyl estradiol and levonorgestrel was supported based on comparable retention times between standards and samples. However, levonorgestrel is the biologically active enantiomer [18] of norgestrel, a racemic mixture of levonorgestrel (d-norgestrel, (−)-norgestrel) and dextronorgestrel (l-norgestrel, (+)-norgestrel) [25, 26], and is indistinguishable from norgestrel with the achiral chromatographic methods used. Norgestrel can be available in various contraceptive tablets. To verify the suspect sample was not norgestrel (thus containing half the amount of levonorgestrel), chiral chromatography available for identifying and quantifying dextronorgestrel in levonorgestrel tablets was applied to both samples (Figure 2). Although complete baseline separation of levonorgestrel and dextronorgestrel was not fully achieved (making dextronorgestrel quantification difficult), enough separation of the enantiomers occurred to indicate the suspect sample was essentially levonorgestrel, with no indication of any significant level of dextronorgestrel.

### 3.4 Spectral comparison

**Figure 3:**
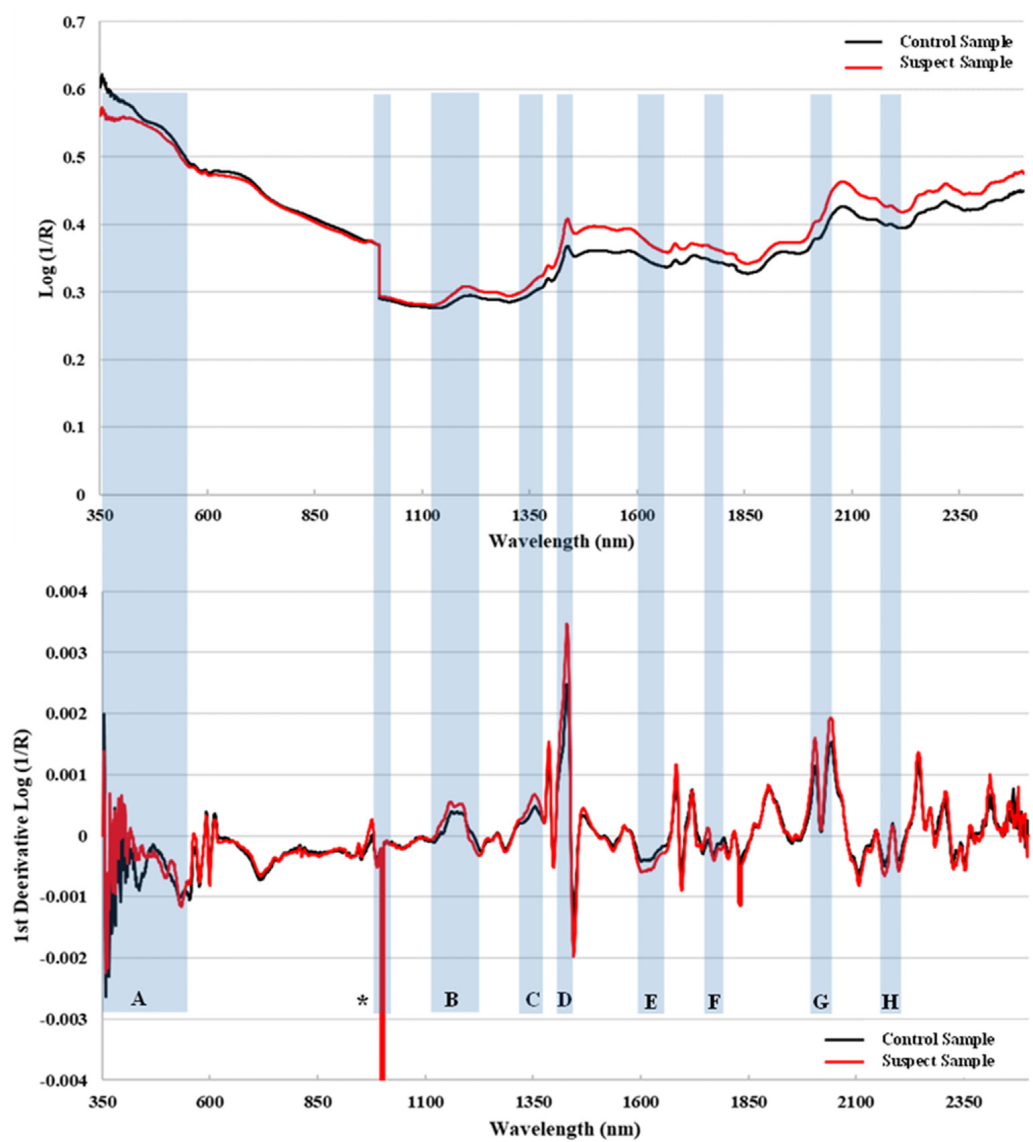
Diffuse reflectance spectra of the control and suspect samples in both Log (1/R) and 1st derivative of Log (1/R) with proposed band assignments: A (∼350–550 nm) = color differences; B (∼1120–1230 nm) = C–H stretch [27, 28]; C (∼1320–1390 nm) = C–H deformation [27, 28]; D (∼1420–1430 nm) = O–H deformation [28, 29]; E (∼1600–1660 nm) = C–H stretch (possibly aliphatic) [27, 28]; F (∼1770–1800 nm) = C–H stretch (possibly cellulosic) [27, 28]; G (∼2010–2045 nm) = O–H stretch/bend combination [29]; H (∼2160–2200 nm) = O–H/C–O stretch combination [30]; ∗ sharp baseline shift associated with detector change at 1000 nm.

Comparison of the diffuse reflectance spectra (covering visible and NIR) indicated differences in the suspect sample relative to the authentic sample. Lower Mahalanobis distances (D) indicate a closer spectral agreement with the authentic product. The control sample was within the D_max_ for the method, while the suspect sample was well outside the limit. The exact source of these differences cannot be determined from the spectra, but could be due to differences in excipients, particle size, moisture and/or hardness. Figure 3 provides the diffuse reflectance spectra for the control and suspect samples. From Log (1/R) spectra, the lower absorbance (higher reflectance) observed at 350–550 nm for the suspect sample appears consistent with the lighter color, where the overall baseline shift may be from particle size and/or hardness differences. The 1st derivative spectra further highlight various regions where differences can be observed that may arise through various O–H and C–H absorptions from aliphatic and carbohydrate components.

## 4 Discussion

Although this falsified product had appropriate levels of active ingredient and dissolution, the reflectance spectrum was distinguishable from the authentic product, which was consistent with the visual assessments. There is no indication that the suspect sample is the authentic product, however the sample appears to be made by a source that is experienced in the production of hormonal contraceptive tablets. However, this does not alleviate the potential risk for this sample. The sample may not have been deemed suspect if only the chemical tests presented here were relied upon. For this work, the visual and diffuse reflectance spectroscopy assessments were vital to the sample analysis and highlights the need for multi-faceted approaches to characterize suspect samples. The results from this analysis were provided to USAID and the manufacturer so that necessary legal and regulatory actions could be pursued. With this sample being presented as another registered product’s brand name, the integrity of the regulatory oversight that the end users rely upon was compromised and points to the continued need to further assist regulatory programs in the global market.

With an end user’s reliance on accurate packaging and labeling, this research highlights the potential risk for other product suppliers that may attempt to present their product (which may be substandard) as that of another well-known brand. Furthermore, the prominence of this sample is unknown in the general market regarding the number of blister packs available or the number of times this has occurred with other products. Although dissolution levels appeared adequate, the clinical level of bioequivalence of this suspect sample relative to the product provided by USAID is unclear. Additionally, there could be other tablets in the market that may have no active ingredient.

Utilization of quality medicines is important for universal health coverage [31, 32] due to the multifaceted problems created from poor quality medicines, such as increased risk of further sickness, deaths, and resistance to the medication [33]. Substandard and falsified medicines could be on the order of 25% within various markets [34], risking increases with product resistance [35] and adverse events [36]. Continued vigilance for enhanced interventions is needed [37] while improving infrastructure coordination [38]. Field surveys are often required for detection with provisions for appropriately handling the ethical challenges associated product acquisition and results dissemination [39]. As supported from this research, incorporating visual assessments [40] and other techniques (i.e., spectroscopic and/or various field portable options [11]) for screening are valuable tools for detecting poor quality medicines, provided pharmacopeia techniques are included to provide a holistic understanding for appropriate action through regulatory authorities.

## Declarations

### Author contribution statement

David Jenkins: Conceived and designed the experiments; Analyzed and interpreted the data; Contributed reagents, materials, analysis tools or data; Wrote the paper.

Cherif Diallo, Michael Payne: Performed the experiments; Analyzed and interpreted the data; Contributed reagents, materials, analysis tools or data; Wrote the paper.

### Funding statement

This work was supported by 10.13039/100000200United States Agency for International Development (AID-OAA-C-15-00001).

### Data availability statement

Data included in article/supplementary material/referenced in article.

### Declaration of interests statement

The authors declare no conflict of interest.

### Additional information

No additional information is available for this paper.
